# Integrated multi-omics analysis to explore correlations among cord blood IGF-1 levels, metabolomic profiles, and meconium gut microbiota in small-for-gestational-age neonates

**DOI:** 10.3389/fped.2026.1942176

**Published:** 2026-09-14

**Authors:** Yuanlong Li, Haizhen Wang, Liangying Luo, Junwei Xie, Yumei Duan, Junqiong Wang, Yan Huang

**Affiliations:** Department of Paediatrics, Hainan Women and Children’s Medical Centre, Haikou, Hainan, China

**Keywords:** 16S rRNA microbiota sequencing, cord blood metabolome, gut microbiota, insulin-like growth factor-1, small for gestational age

## Abstract

**Background:**

Small for gestational age (SGA) infants carry high risks of neonatal complications and lifelong metabolic disorders. This study explored correlations among cord blood IGF-1, cord blood metabolome, and meconium microbiota in SGA neonates to clarify the underlying pathophysiology.

**Methods:**

We consecutively recruited 113 term newborns (53 SGA, 60 AGA) delivered at Hainan Women and Children's Medical Centre from June 2024 to March 2025. Cord blood IGF-1 was tested via chemiluminescence. UHPLC-MS/MS untargeted metabolomics and 16S rRNA high-throughput sequencing were applied to characterize cord blood metabolites and meconium microbiota. Intergroup differences and partial Spearman correlations across IGF-1, metabolites, microbes and birth indices were analyzed, adjusting for gestational age, infant sex, and maternal delivery mode,where applicable.

**Results:**

(1) SGA infants had markedly lower cord blood IGF-1 (*P* < 0.001), which positively correlated with birth weight and length. (partial Spearman *r* = 0.634 and 0.494, respectively, *P* < 0.001, after adjusting for gestational age, sex, and maternal BMI). (2) Two groups presented distinct metabolic profiles. SGA infants showed depleted glycerophospholipids, elevated chlorobenzene pollutants, and altered pathways involving bile secretion, choline and steroid metabolism. (3) No alpha diversity difference existed, yet beta diversity confirmed divergent microbial structures (R^2^ = 0.0284, *P* = 0.046). SGA overrepresented *Clostridium* and *Klebsiella*, with reduced facultative pioneer bacteria. (4) SGA-enriched anaerobes negatively correlated with birth size and IGF-1, while chlorobenzene levels were positively associated with these pathogenic genera.

**Conclusions:**

SGA neonates feature suppressed IGF-1, intrauterine metabolic disorders and disordered initial gut colonization. Disturbed prenatal metabolism and pollutant exposure may jointly drive aberrant early microbiota development in growth-restricted newborns. The cross-sectional design precludes causal inference, and the chlorobenzene finding warrants independent validation.

## 1 Introduction

Small for gestational age (SGA) refers to newborns whose birth weight or length is below the 10th percentile for newborns of the same gestational age and sex. Not only does SGA increase the risk of short-term neonatal complications such as hypoglycemia, polycythemia, and necrotizing enterocolitis, but it also acts as a risk factor for long-term metabolic syndromes including obesity, type 2 diabetes, and cardiovascular diseases in childhood and adulthood (1–4). Therefore, in-depth elucidation of the pathophysiological mechanisms underlying the occurrence and progression of SGA holds significant public health and clinical importance.

Within the regulatory network of fetal growth and development, the growth hormone-insulin-like growth factor-1 (GH-IGF-1) axis plays a central regulatory role. IGF-1 is a key endocrine molecule mediating fetal cell proliferation, tissue differentiation, and energy metabolism. Cord blood IGF-1 levels objectively reflect fetal intrauterine nutritional reserves and growth potential, and its downregulation is a typical endocrine phenotype of SGA (5–7). In recent years, the application of metabolomics technology has further revealed that SGA fetuses undergo systematic intrauterine metabolic reprogramming, wherein insufficient synthesis of lipid molecules such as glycerophospholipids is considered the core material basis restricting fetal tissue growth (8–10). Meanwhile, a growing body of epidemiological evidence indicates that prenatal exposure to environmental chemicals can cross the placental barrier into the fetal circulation, disrupt normal developmental processes, and serve as a potential non-nutritional risk factor affecting intrauterine fetal growth (11, 12). However, the interactive regulatory relationships among intrauterine metabolic abnormalities, environmental pollutant exposure, and the IGF-1 endocrine axis, as well as their combined mechanisms on fetal growth, remain to be systematically elucidated.

The perinatal period represents a critical window for gut microbiota colonization and microecosystem establishment in early life. Normal microbiota colonization not only participates in nutrient digestion and absorption but is also an essential prerequisite for the development and maturation of the intestinal mucosal immune barrier (13). Accumulating evidence has demonstrated that due to intrauterine growth restriction, SGA newborns exhibit relatively immature intestinal tissue development and frequently manifest microecological imbalance characterized by disordered microbiota colonization timing and enrichment of opportunistic pathogens in the early postnatal period. Such early microbiota alterations are considered one of the potential mediators of adverse outcomes in SGA infants (14–16). However, current research on SGA has predominantly focused on either microbiota or metabolic characteristics in isolation, without integrating the IGF-1 endocrine axis, intrauterine metabolic profiles, and early gut microbiota for comprehensive analysis. The mechanisms by which intrauterine metabolic abnormalities and environmental exposures synergistically influence early microbiota colonization in SGA remain unclear.

Therefore, this study aims to integrate cord blood IGF-1 detection, untargeted cord blood metabolomics analysis, and high-throughput 16S rRNA sequencing of meconium to systematically compare the differences in endocrine, metabolomic, and microbiome profiles between small for gestational age (SGA) and appropriate for gestational age (AGA) infants, and analyze their correlations with birth physical parameters. It further explores associations between the intrauterine metabolic environment and endocrine status and early gut microbiota colonization in SGA infants. This study aims to enrich the understanding of SGA pathophysiology from an integrated “endocrine-metabolic-microecological” perspective, providing a theoretical basis for subsequent development of early risk identification and microecological intervention strategies.

## 2 Materials and methods

### 2.1 Study subjects

This study was a cross-sectional study. All eligible newborns born in the Department of Obstetrics at Hainan Women and Children Medical Center from June 2024 to March 2025 were consecutively enrolled as study subjects. According to birth weight below the 10th percentile (P10) for newborns of the same gestational age and sex, they were divided into the SGA group and the appropriate for gestational age (AGA) group. A total of 113 neonates were enrolled, comprising 53 SGA and 60 AGA infants. Meconium samples were collected from 108 neonates (51 SGA and 57 AGA) within 48 h after birth; all 113 neonates provided cord blood samples.

Inclusion criteria: (1) Live-born newborns delivered within the specified timeframe; (2) Written informed consent was signed by the parents or guardians of the newborn.

Exclusion criteria: (1) Uncertain gestational age; (2) Death at birth; (3) Inability to complete weight measurement within 24 h after birth; (4) Presence of diseases affecting physical measurement indicators, such as severe congenital malformations, genetic metabolic diseases, or fetal hydrops; (5) Maternal severe underlying diseases (e.g., severe heart disease, kidney disease, autoimmune diseases, active infection, malignant tumors, etc.).

This study was approved by the Ethics Committee of Hainan Women and Children Medical Center (Ethical Approval Number: HNWCMC).

### 2.2 Clinical data and sample collection

General data of the newborns at birth were collected, including sex, birth weight, length, head circumference, gestational age, maternal BMI, maternal age, delivery mode, pregnancy complications (gestational diabetes mellitus, hypertension, cholestasis, etc.), and medication use (antibiotics, steroid hormones, etc.). Meconium samples were collected within 48 h after birth, placed in sterile cryopreservation tubes containing fixative, and immediately stored in an ultra-low temperature freezer at −80°C for later use. Simultaneously, 4 ml of cord blood was collected at birth. After processing by low-temperature centrifugation (3,000 rpm, 4°C, 10 min), the supernatant was aspirated and aliquoted into sterile cryopreservation tubes at 1 ml per tube. These samples were likewise stored in an ultra-low temperature freezer at −80°C for subsequent experimental analyses.

### 2.3 Sample preparation and data analysis

#### 2.3.1 Insulin-like growth factor-1 (IGF-1) detection

Cord blood IGF-1 levels in the two groups were measured by chemiluminescence, strictly following the manufacturer's instructions for the detection kit (Siemens Medical Diagnostics Products Ltd., UK) to ensure the accuracy and reliability of the test results.

#### 2.3.2 Cord blood metabolomics sample preparation and Lc-Ms/Ms analysis

##### 2.3.2.1 Sample extraction and QC construction

100 µl of cord blood serum was added to a 1.5 ml EP tube, followed by the addition of 400 µl of pre-cooled extraction buffer (acetonitrile:methanol = 1:1, v/v, containing 0.02 mg/ml L-2-chlorophenylalanine as an internal standard). The mixture was vortexed for 30 s, sonicated under conditions of 5°C and 40 kHz for 30 min, and then incubated at −20°C for 30 min to precipitate proteins. After centrifugation at 13,000 × g at 4°C for 15 min, the supernatant was collected and dried under a stream of nitrogen. The residue was reconstituted in 100 µl of resuspension solution (acetonitrile:water = 1:1, v/v), subjected to ultrasonic extraction at 5°C for 5 min, and centrifuged again at 13,000 × g at 4°C for 10 min. The resulting supernatant was transferred into sample vials for injection. Meanwhile, a quality control (QC) sample was prepared by pooling equal volumes of aliquots from all samples to monitor the stability of the analytical process. One QC sample was injected every 5–10 samples during the instrumental run. Extraction blanks (solvent only) were processed in parallel to monitor for background contamination.

##### 2.3.2.2 LC-MS/MS detection

Data acquisition was performed using a Thermo UHPLC-Orbitrap Exploris 240 high-resolution mass spectrometry system. Chromatographic separation was carried out on a Waters ACQUITY UPLC HSS T3 column (100 mm × 2.1 mm, 1.8 µm). The mobile phase consisted of solvent A (water/acetonitrile = 95:5, v/v, containing 0.1% formic acid) and solvent B (acetonitrile/isopropanol/water = 47.5:47.5:5, v/v/v, containing 0.1% formic acid). The flow rate was set at 0.40 ml/min, and the column temperature was maintained at 40°C. Mass spectrometry data were acquired in positive/negative ion switching mode with a scan range of m/z 70–1,050. Ionization parameters were set as follows: spray voltage at +3,500 V for positive mode and −3,000 V for negative mode; sheath gas flow rate at 50 arb; auxiliary gas flow rate at 13 arb; capillary temperature at 450°C; and collision energy applied in a stepped mode of 20–40–60 V.

##### 2.3.2.3 Metabolomics data processing and analysis

Raw LC-MS files were imported into Progenesis QI software (Waters Corporation, Milford, USA) for baseline filtering, peak picking, integration, retention time correction, and peak alignment to construct a three-dimensional data matrix comprising retention time, mass-to-charge ratio (m/z), and peak intensity. Metabolite identification was achieved by matching MS/MS fragmentation patterns against the HMDB (http://www.hmdb.ca/), METLIN (https://metlin.scripps.edu/), and an in-house standard library, achieving Metabolomics Standards Initiative (MSI) Level 2 (putative identification by spectral library matching) confidence. No Level 3 identifications (confirmed by co-elution with authentic chemical standards) were obtained for the reported differential metabolites. Among the 1,734 detected metabolic features, the 331 significantly differential metabolites reported were those with Level 2 identification confidence. To eliminate missing values and random errors, the 80% rule was applied for variable filtering (retaining metabolites with non-zero values in >80% of samples within at least one group, applied globally across all samples). Missing values were imputed with the minimum observed value in the raw data matrix. Subsequently, sum normalization was performed to correct signal deviations caused by sample preparation and instrumental fluctuations. Metabolites with a relative standard deviation (RSD) >30% in QC samples were excluded, and data were log10-transformed to approximate a normal distribution, yielding a clean data matrix for statistical analysis. A supervised orthogonal partial least squares discriminant analysis (OPLS-DA) model was constructed using the ropls package (v1.6.2) in R. Model robustness was validated by 200-response permutation testing. Differential metabolites were defined as those satisfying all three of the following criteria: variable importance in projection (VIP) >1 based on the OPLS-DA model, Student's *t*-test *P* < 0.05, and false discovery rate (FDR) <0.05 using the Benjamini-Hochberg method. Pathway annotation was performed using the KEGG database (https://www.kegg.jp/), and Fisher's exact test (implemented in the Python SciPy.stats module) was employed to identify significantly enriched metabolic pathways (*P* < 0.05).

#### 2.3.3 Detection and bioinformatics analysis of gut microbiota

##### 2.3.3.1 DNA extraction and quality control

Total genomic DNA of the microbial community in fecal samples was extracted according to the manufacturer's instructions of the E.Z.N.A.® Soil DNA Kit (Omega Bio-tek, Norcross, GA, USA). The integrity of the extracted DNA was verified by 1% agarose gel electrophoresis, and its concentration and purity were measured using a NanoDrop 2000 spectrophotometer (Thermo Scientific, Wilmington, DE, USA) to ensure that the DNA quality met the requirements for subsequent library construction. Negative controls, including extraction blanks and PCR no-template controls, were processed in parallel and sequenced to monitor potential environmental or reagent contamination.

##### 2.3.3.2 PCR amplification and sequencing library construction

Using qualified genomic DNA as the template, the V3–V4 hypervariable regions of the bacterial 16S rRNA gene were amplified with barcode-labeled specific primers. The forward primer was 338F (5′-ACTCCTACGGGAGGCAGCAG-3′) and the reverse primer was 806R (5′-GGACTACHVGGGTWTCTAAT-3′). The total volume of the PCR reaction system was 20 µl, comprising 10 µl of 2×Pro Taq Master Mix (Accurate Biology, Hunan, China), 0.8 µl each of the forward and reverse primers (5 µm), 10 ng of template DNA, and the remaining volume supplemented with sterile deionized water. The amplification program was set as follows: initial denaturation at 95°C for 3 min; followed by 27 cycles of denaturation at 95°C for 30 s, annealing at 55°C for 30 s, and extension at 72°C for 45 s; with a final extension at 72°C for 10 min. The products were stored at 10°C. After separation by 2% agarose gel electrophoresis, the amplification products were purified by gel excision using the SanPrep Column DNA Gel Extraction Kit (Sangon Biotech, Shanghai, China). Following accurate quantification of the purified products using a Qubit 4.0 Fluorometer (Thermo Fisher Scientific, Waltham, MA, USA), libraries were constructed using the NEXTFLEX® Rapid DNA-Seq Kit. After passing quality inspection, the constructed libraries were entrusted to Shanghai Majorbio Bio-pharm Technology Co., Ltd. for paired-end high-throughput sequencing on the Illumina Nextseq 2000 platform.

##### 2.3.3.3 Bioinformatics analysis

Raw sequencing data were subjected to rigorous quality control, denoising, assembly, and chimera filtering to obtain high-quality effective sequences (Clean Tags). Qualified clean reads were clustered into operational taxonomic units (OTUs) at a 97% nucleotide similarity threshold using the USEARCH v11 algorithm. An OTU is defined as a cluster of sequencing reads sharing ≥97% sequence identity, which served as the unified taxonomic unit for all downstream analyses. Taxonomic classification of each representative OTU sequence was performed against the SILVA 138.2 database. Subsequent bioinformatics analyses were performed on the Majorbio Cloud Platform (https://cloud.majorbio.com) and the SangerBox Cloud Platform (https://cloud.i-sanger.com): The Chao1 richness index and Shannon diversity index were calculated using Mothur software, and intergroup differences in alpha diversity were compared by the Wilcoxon rank-sum test. Principal coordinate analysis (PCoA) based on the Bray-Curtis distance algorithm was conducted to visually illustrate the similarity and heterogeneity of microbial community structures among samples. Meanwhile, the statistical significance of intergroup differences in community structure was assessed by permutational multivariate analysis of variance (PERMANOVA, Adonis). PERMANOVA was additionally performed with delivery mode, gestational age, and sex as covariates to adjust for potential confounding effects. The linear discriminant analysis effect size (LEfSe) algorithm was employed to identify differentially abundant taxa between groups, with a linear discriminant analysis (LDA) score threshold set at >2 and a *P*-value <0.05. Ultimately, bacterial taxa exhibiting significant differences in genus-level abundance between the SGA and AGA groups were identified.

### 2.4 Statistical analysis

GraphPad Prism 10.0 and SPSS 29.0 statistical software were used for figure generation and data analysis. Continuous variables were presented as mean ± standard deviation (x ± s) if normally distributed, or as median (M) and interquartile range (P25, P75) if not normally distributed. Normality was assessed using the Shapiro–Wilk test. Comparisons of continuous variables between the two groups were performed using the independent-samples *t*-test for normally distributed data and the Mann–Whitney *U*-test for non-normally distributed data. Microbiota relative abundance data, which are inherently bounded and non-normal, were analyzed using the Mann–Whitney *U*-test or Wilcoxon rank-sum test. Categorical variables were expressed as percentages (%), and comparisons between groups were conducted using the chi-square (χ^2^) test or Fisher's exact test where appropriate. Partial Spearman correlation, adjusting for gestational age, sex, and maternal delivery mode, was used to assess the independent associations between IGF-1, birth parameters, differential metabolites, and microbial taxa. A sensitivity analysis excluding neonates with maternal pre-delivery antibiotic exposure was additionally performed for microbiome outcomes. A *P*-value <0.05 was considered statistically significant.

## 3 Results

### 3.1 Clinical characteristics analysis

A total of 113 newborns born at our hospital (Hainan Women and Children Medical Center) from June 2024 to March 2025 were enrolled in this study and divided into the AGA group (*n* = 60) and SGA group (*n* = 53) based on birth weight. The 5 missing meconium samples were distributed as 2 in the SGA group (1 due to failure to pass meconium within 48 h, 1 due to parental refusal) and 3 in the AGA group (2 due to delayed meconium passage, 1 due to insufficient sample volume). There was no statistically significant difference in attrition rates between groups (Fisher's exact test, *P* = 0.367). Cord blood samples were obtained from all 113 cases. Statistically significant differences were observed in birth weight and birth length between the AGA and SGA groups (*P* < 0.05). No statistically significant differences were found between the two groups in terms of gestational age at birth, delivery mode, sex, maternal age, pre-delivery maternal BMI, pre-delivery use of steroid hormones and antibiotics, or incidences of pregnancy complications including gestational anemia, gestational hypertension, and gestational cholestasis (*P* > 0.05). The incidence of gestational diabetes mellitus in mothers was higher in the AGA group than in the SGA group, with a statistically significant difference (*P* < 0.05) (Table 1). Because gestational diabetes mellitus differed significantly between groups, subsequent multivariate analyses were adjusted for this covariate where applicable.

**Table 1:** Comparison of clinical characteristics between AGA group and SGA group.

Indicator	AGA group (*n* = 60)	SGA group (*n* = 53)	t/X^2^ value	*P*-value	Effect size
Fetus					
Gestational age (weeks)	38.80 ± 1.11	38.51 ± 1.19	1.046	0.298	d = 0.25
Birth weight (g)	3,245.00 (2947.5, 3477.5)	2,580.00 (2,395.00, 2,705.00)	10.993	<0.001	d = 2.10
Birth length (cm)	50.00 (49.00, 50.00)	47.00 (46.00, 48.00)	9.965	<0.001	d = 1.52
Sex (Male/Female), *n*(%)	30 (50.00)/30 (50.00)	29 (54.72)/24 (45.28)	0.098	0.755	OR = 1.21
Maternal					
Maternal age (years)	30.33 ± 4.39	30.30 ± 5.24	0.035	0.972	d = 0.01
Delivery mode					
Vaginal delivery [*n*(%)]	33 (54.10%)	27 (51.92%)	−0.428	0.67	OR = 1.09
Cesarean section [*n*(%)]	28 (45.90%)	25 (48.08%)			
Maternal pre-delivery BMI (kg/m^2^)	26.98 ± 3.19	25.85 ± 3.80	1.727	0.087	d = 0.32
Gestational anemia [*n*(%)]	13 (21.31%)	10 (19.23%)	0.366	0.715	OR = 1.14
Gestational diabetes mellitus [*n*(%)]	13 (21.31%)	4 (7.69%)	2.118	0.036	OR = 3.25
Gestational hypertension [*n*(%)]	4 (6.56%)	3 (5.77%)	0.22	0.827	OR = 1.15
Gestational cholestasis [*n*(%)]	0 (0.00%)	2 (3.85%)	−1.52	0.131	–
Pre-delivery steroid hormones [*n*(%)]	0 (0.00%)	1 (1.92%)	−1.066	0.289	–
Pre-delivery antibiotic use [*n*(%)]	7 (11.48%)	5 (9.62%)	0.347	0.729	OR = 1.22

d, Cohen's d; OR, odds ratio. Effect sizes are provided for variables with significant or near-significant differences.

### 3.2 Analysis of neonatal cord blood IGF-1 levels and their correlation with clinical characteristics

Cord blood IGF-1 levels in the SGA group were significantly lower than those in the AGA group [27.8 (21.16, 38.73) ng/ml vs. 49.72 (30.10, 60.08) ng/ml, *Z* = 5.929, *P* < 0.001]. Spearman correlation analysis showed that cord blood IGF-1 levels were significantly positively correlated with neonatal birth weight (*r* = 0.562, *P* < 0.001) and birth length (*r* = 0.465, *P* < 0.001). Partial Spearman correlation analysis, adjusted for gestational age, maternal BMI and neonatal sex, revealed that cord blood IGF-1 levels were significantly positively correlated with birth weight (*r* = 0.634, *P* < 0.001) and birth length (*r* = 0.494, *P* < 0.001). The corresponding coefficients of determination (r^2^) indicated that IGF-1 explained approximately 40.2% of the variance in birth weight and 24.4% of the variance in birth length, suggesting that IGF-1 explains only a modest proportion of the variance in fetal growth parameters, with the majority attributable to other genetic, nutritional, and environmental factor.

### 3.3 Cord blood metabolomic analysis of SGA and AGA newborns

#### 3.3.1 Overall characteristics of cord blood metabolomics

A total of 1734 metabolites were identified in the cord blood samples from both groups. To assess the overall distribution characteristics of the cord blood metabolome between the two groups, OPLS-DA analysis was performed. The results showed that although there was some individual heterogeneity within groups, the cord blood samples from SGA and AGA exhibited a separation trend along the first two principal components, indicating the presence of systemic metabolic characteristic differences between the two groups. The model cumulatively explained 66.4% of the intergroup variation [R^2^Y(cum) = 0.664] and had a cumulative predictive ability of 0.474 [Q^2^(cum) = 0.474]. Furthermore, the Q^2^ intercept of the model after 200 permutation tests was −0.2146, confirming that the model was not overfitted and that the metabolomic separation between the two groups was statistically significant (Figure 1). Notably, Q^2^(cum) of 0.474 falls slightly below the conventional threshold of 0.5 for strong predictive relevance, suggesting that while statistically significant, the model's predictive power is modest and should be interpreted with caution.

**Figure 1:**
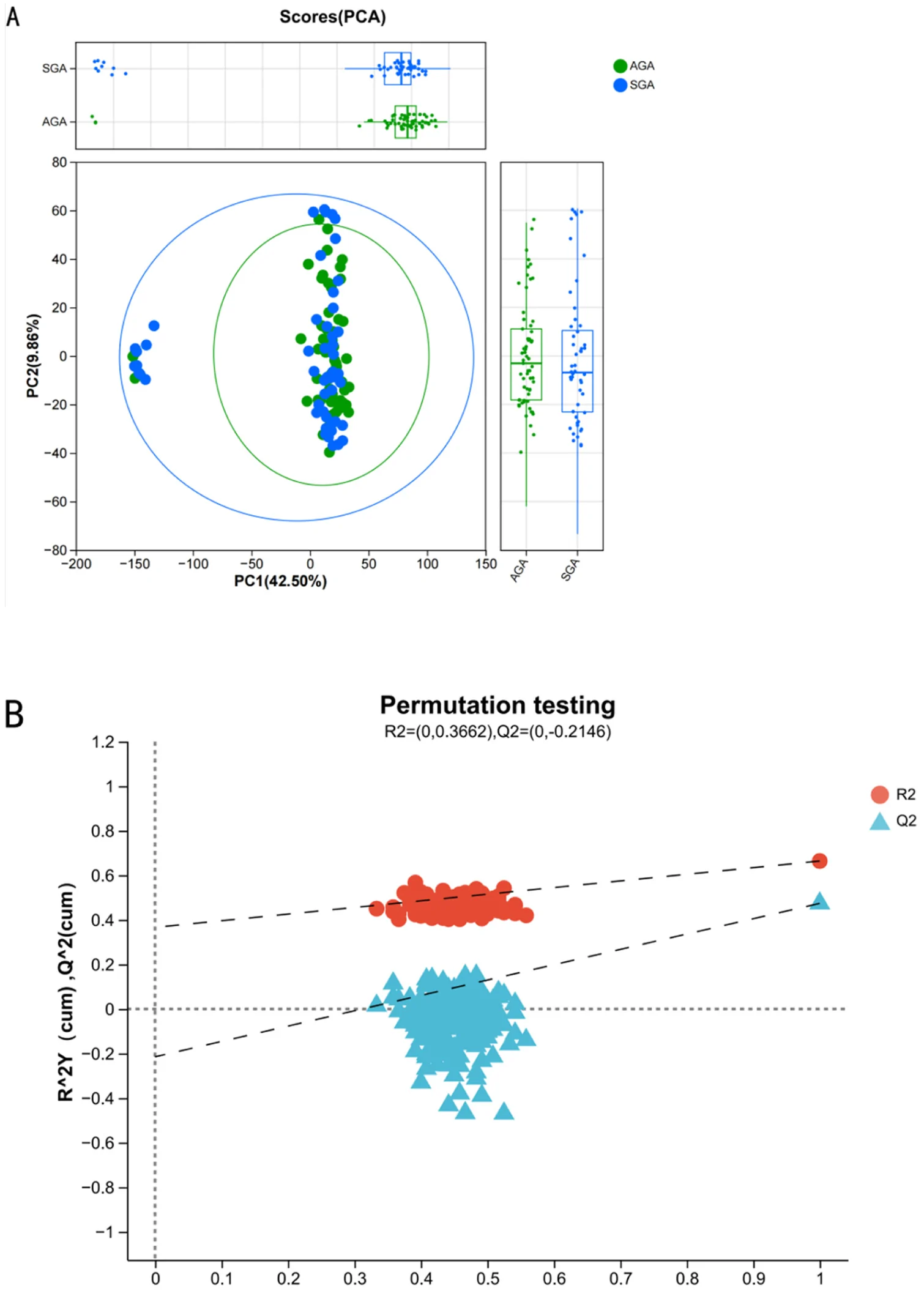
OPLS-DA analysis of cord blood metabolomes in SGA vs AGA neonates. **(A)** Score plot of the first two principal components based on OPLS-DA analysis. **(B)** Results of permutation testing. The red circles represent the regression line for R^2^ (intercept = 0.3662), and the blue triangles represent the regression line for Q^2^ (intercept = −0.2146). The negative intercept of the Q^2^ regression line confirms that the OPLS-DA model is robust.

#### 3.3.2 Analysis of differential metabolites in cord blood metabolome between the two groups

Based on the OPLS-DA model and predefined statistical screening criteria (*P* < 0.05, FDR<0.05, and variable importance in projection value VIP_pred_OPLS-DA >1), a total of 331 significantly differentially expressed metabolites were identified in the cord blood samples between small for gestational age (SGA) and appropriate for gestational age (AGA) infants. Among them, 229 metabolites were significantly downregulated in the SGA group. The downregulated metabolites were primarily lipids and lipid-like molecules (accounting for approximately 65%), with glycerophospholipids being the most significantly decreased subclass. This subclass included phosphatidylcholines [e.g., Pc(p-16:0/2:0), Pc(22:5/0:0), Pc(20:3/0:0)], lysophosphatidylcholines [e.g., Lpc(16:1), Lpc(20:4)], and lysophosphatidylethanolamines [e.g., Lpe(22:5)]. This was followed by steroid and steroid derivative subclasses (e.g., cucurbitacin B, aldosterone, dehydroepiandrosterone sulfate, testosterone sulfate) and fatty acids and acyl carnitines (e.g., glutarylcarnitine, 3-hydroxyocta-3,6-dienoylcarnitine, elaidic acid). Additionally, 102 metabolites were significantly upregulated in the SGA group. The upregulated metabolites were predominantly organic nitrogen compounds (accounting for approximately 20%), including amine subclasses (e.g., N-ethylaniline, the xenobiotic compound chlorobenzene, pirimicarb) and guanidine subclasses (e.g., chloroguanide, an antimalarial drug metabolite). The identification of chlorobenzene was based on MS/MS fragmentation pattern matching against the METLIN and in-house standard libraries; however, we acknowledge that chlorobenzene is a common chromatographic contaminant. Rigorous blank control analysis is required to confirm this as a genuine in utero exposure rather than an analytical artifact (Figure 2).

**Figure 2:**
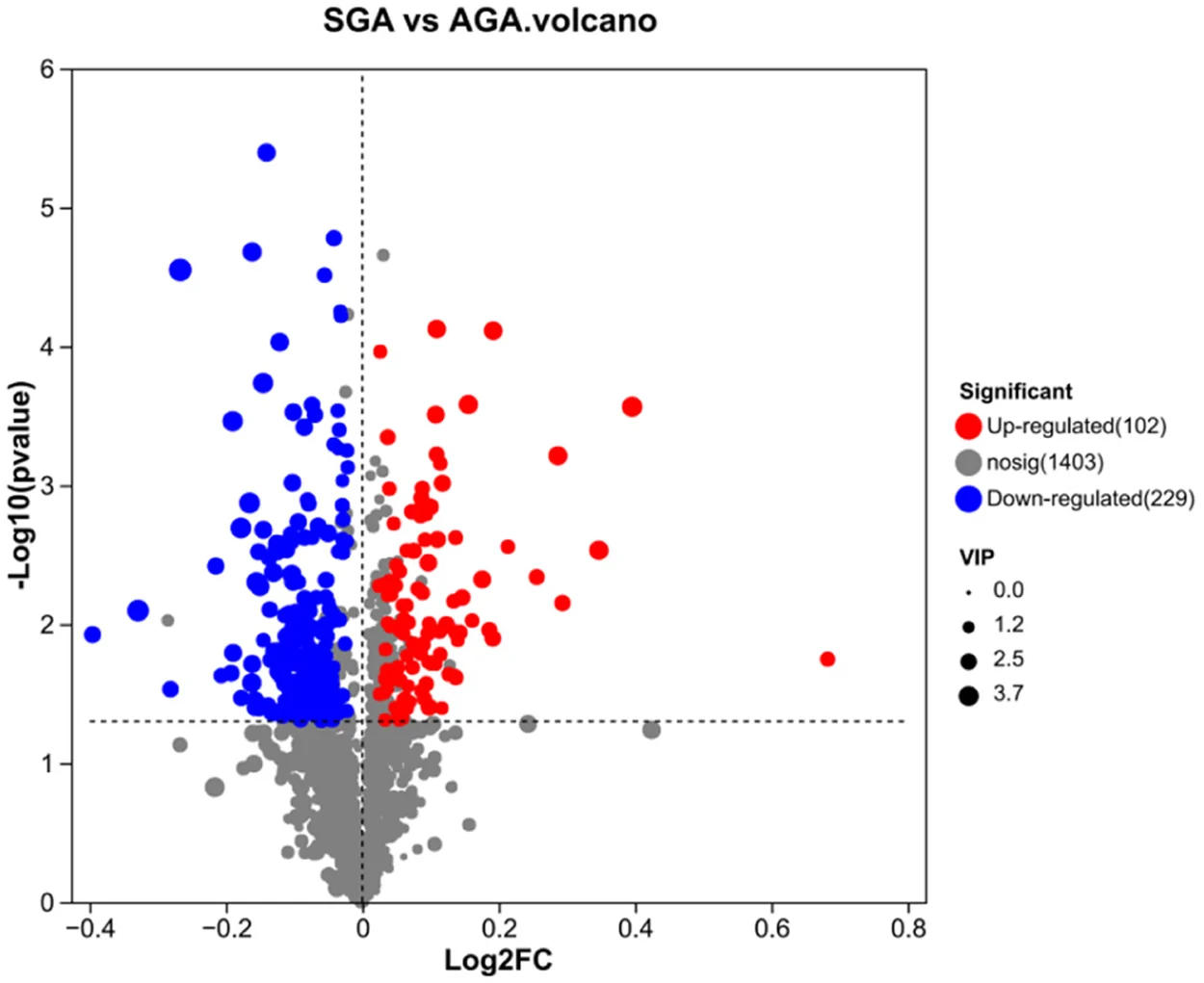
Volcano plot of differential metabolites in cord blood between SGA and AGA groups. Red dots indicate significantly up-regulated metabolites in SGA; blue dots indicate significantly down-regulated metabolites in SGA; gray dots indicate non-significant metabolites.

#### 3.3.3 Enrichment analysis of differential metabolites in cord blood metabolome between the two groups

Enrichment analysis based on the KEGG database revealed that among the 331 significantly differential metabolites in SGA cord blood, a total of 4 pathways reached statistical significance (FDR-corrected *p* < 0.05) after multiple testing correction (Benjamini-Hochberg method). These specifically included the bile secretion pathway, choline metabolism in cancer pathway, steroid hormone biosynthesis pathway, and arginine biosynthesis pathway (Figure 3).

**Figure 3:**
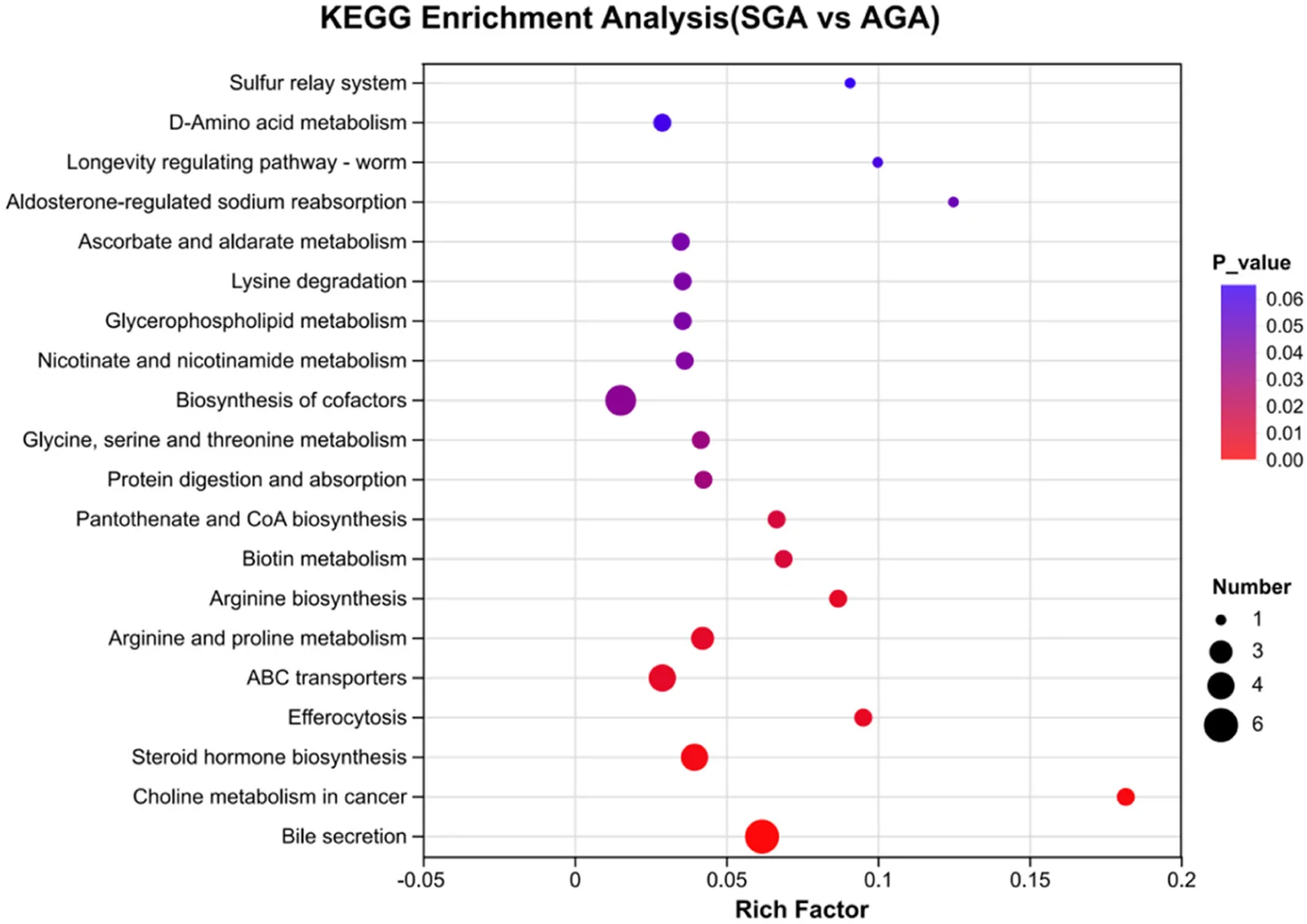
Bubble diagram of KEGG pathway enrichment for differential cord blood metabolites between SGA and AGA groups. The size of each bubble represents the number of metabolites in the pathway; the color represents the FDR-adjusted *P*-value.

### 3.4 Gut microbiota analysis of SGA and AGA newborns

#### 3.4.1 Overall structural differences in gut microbiota

Alpha diversity analysis showed no statistically significant differences in gut microbiota richness (Chao index, *P* = 0.089) or diversity (Shannon index, *P* = 0.346) between the two groups. However, beta diversity analysis (PCoA) revealed a significant difference in the overall community structure of the gut microbiota between the two groups (R^2^ = 0.0285, *P* = 0.048), although the effect size was small, indicating that group membership explains only approximately 2.9% of the variance in community structure After adjusting for delivery mode, gestational age, and sex, the between-group difference in beta diversity remained marginally significant (R^2^ = 0.0284, *P* = 0.046) (Figure 4). In a sensitivity analysis excluding neonates with maternal pre-delivery antibiotic exposure (*n* = 12), the between-group beta diversity difference remained significant (R^2^ = 0.0368, *P* = 0.031), and the core differential genera (*Clostridium, Klebsiella*) identified by LEfSe were consistent with the primary analysis, supporting the robustness of our findings.

**Figure 4:**
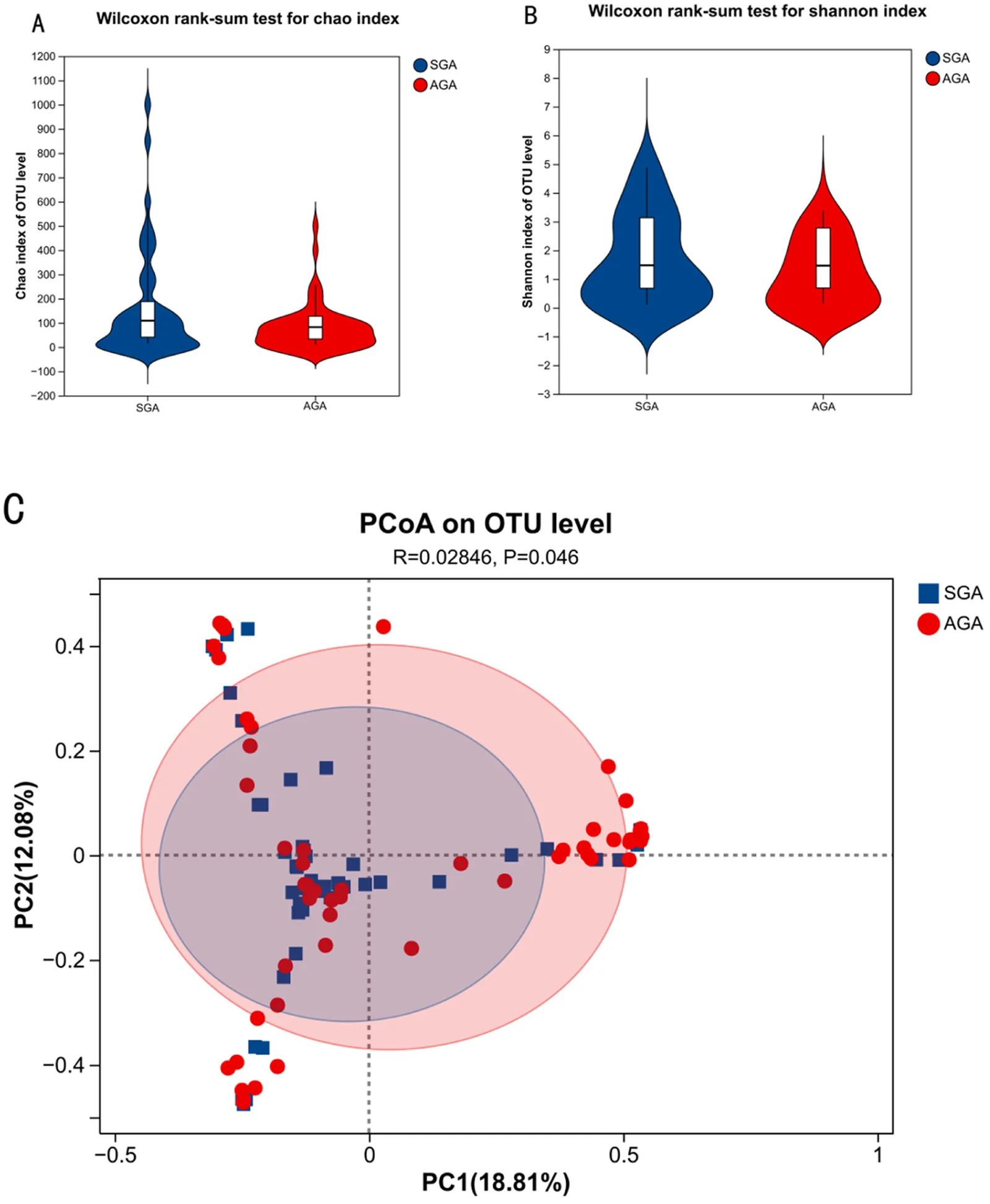
Analysis of gut microbiota structure. **(A)** Comparison of Chao indices of meconium microbiota between the two newborn groups. **(B)** Comparison of Shannon indices of meconium microbiota between the two newborn groups. **(C)** PCoA analysis of meconium microbiota between the two groups at the OTU level.

#### 3.4.2 Overall compositional analysis of gut microbiota

Analysis at the phylum level showed that the microbiota composition in both the AGA and SGA groups was dominated by *Pseudomonadota* (formerly *Proteobacteria*), *Bacillota* (formerly *Firmicutes*), *Actinomycetota*, and *Bacteroidota* (formerly *Bacteroidetes*), which together accounted for over 90% of the total abundance. These synonymies reflect updated GTDB taxonomy; for comparability with prior human microbiome literature, the *Firmicutes/Bacteroidetes* ratio concept corresponds to the *Bacillota/Bacteroidota* ratio in the present nomenclature. In the SGA group, the relative abundance of *Pseudomonadota* decreased from 55.42% to 44.45% compared to the AGA group (*Z* = 1.365, *P* = 0.175), while *Bacteroidota* increased from 6.36% to 10.96% (*Z* = 0.649, *P* = 0.517), and *Bacillota* increased from 26.31% to 31.51% (*Z* = 1.048, *P* = 0.297). No statistically significant differences were observed between the two groups at the phylum level (Figure 5A).

**Figure 5:**
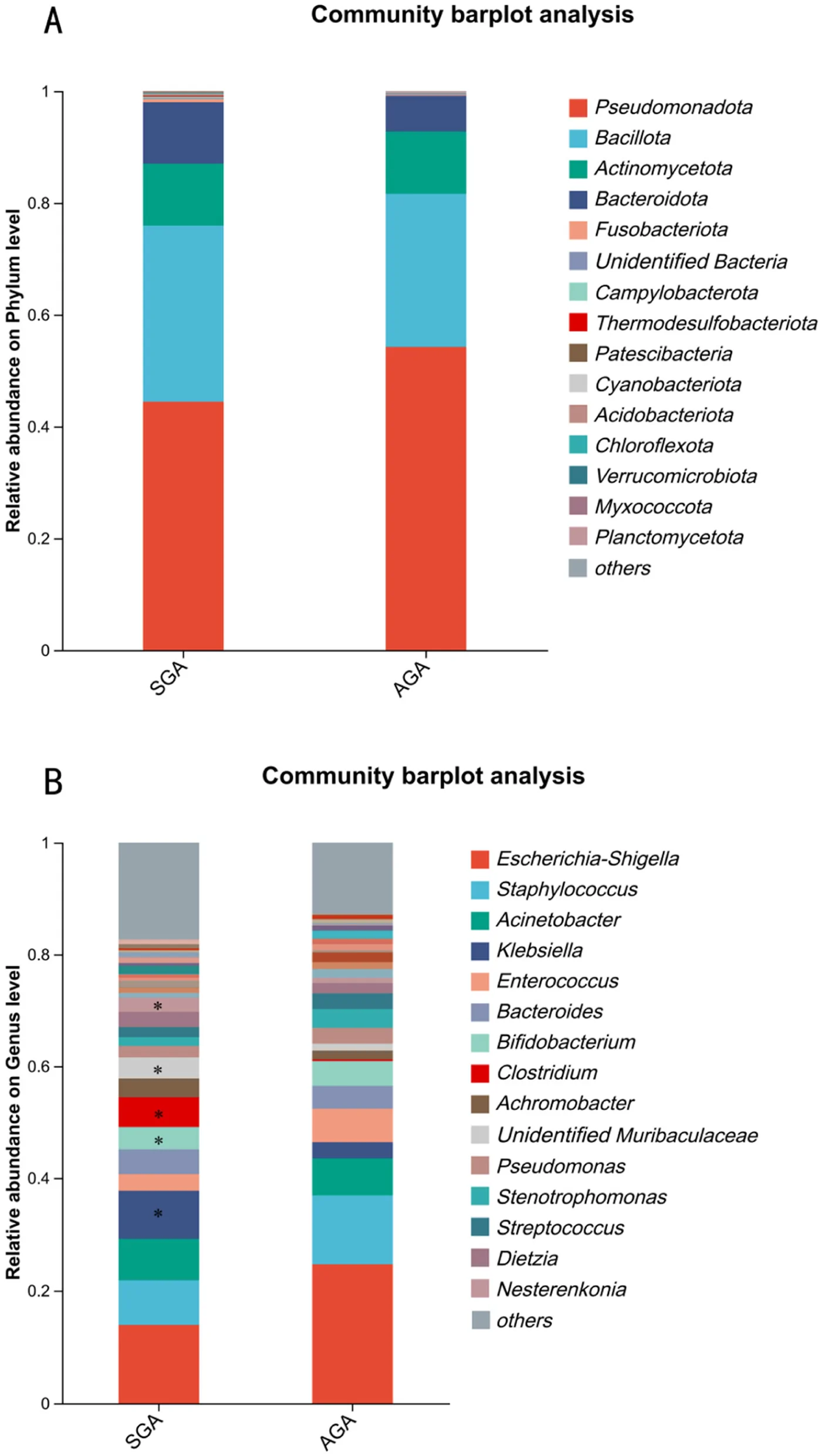
Analysis of gut microbiota composition between the two groups. **(A)** Distribution of gut microbiota composition at the phylum level between the two groups. **(B)** Distribution of gut microbiota composition at the genus level between the two groups. Asterisks (*) denote significant intergroup differences (Mann–Whitney *U*-test, *P* < 0.05).

Analysis at the genus level revealed that the gut microbiota in the AGA group was absolutely dominated by *Escherichia-Shigella* (23.52%), followed by *Staphylococcus* (10.20%), *Acinetobacter* (4.51%), and *Enterococcus* (6.11%). In the SGA group, the relative abundance of *Escherichia-Shigella* decreased from 23.52% to 13.83% (*Z* = 0.280, *P* = 0.790), and *Enterococcus* decreased from 6.11% to 2.94% (*Z* = 0.045, *P* = 0.980). Although these changes did not reach statistical significance, they indicated a trend of overall reduction in these genera. In contrast, the abundances of *Klebsiella* (*Z* = 2.714, *P* = 0.007) and *Clostridium* (*Z* = 3.763, *P* < 0.001) were significantly higher in the SGA group than in the AGA group (Figure 5B).

#### 3.4.3 Identification of intergroup differential microbiota by LEfSe analysis

To identify microorganisms with significant differences at the genus level between the SGA and AGA groups, LEfSe analysis was performed using an LDA score >2.0 and *P* < 0.05 as the screening threshold. A total of 82 differential microbial species with complete grouping information were screened out. Among them, 64 species were significantly enriched in the SGA group (accounting for 78.0%), and 18 species were significantly enriched in the AGA group (accounting for 22.0%). Core genera with significantly increased relative abundance in the SGA group included Clostridium (LDA = 4.38, *P* = 0.0003), *Klebsiella* (LDA = 4.37, *P* = 0.0076), *Clostridioides* (LDA = 3.92, *P* = 0.019), *Nesterenkonia* (LDA = 3.90, *P* = 0.016), and *Blautia* (LDA = 3.55, *P* = 0.038). Core genera with significantly increased relative abundance in the AGA group included *Paraclostridium* (LDA = 3.79, *P* = 0.0025), *Phyllobacterium* (LDA = 3.40, *P* = 0.008), *Roseateles* (LDA = 3.44, *P* = 0.046), *Mycobacterium* (LDA = 3.33, *P* = 0.041), *Aquabacterium* (LDA = 3.30, *P* = 0.031), and *Ruminococcus* (LDA = 3.08, *P* = 0.005) (Figure 6). Notably, *Clostridium, Klebsiella*, and *Blautia* are taxa that can appear as contaminants in low-biomass microbiome studies; however, their enrichment pattern was consistent with the SGA phenotype and was not detected in the sequenced negative extraction blanks, supporting their biological origin in meconium.

**Figure 6:**
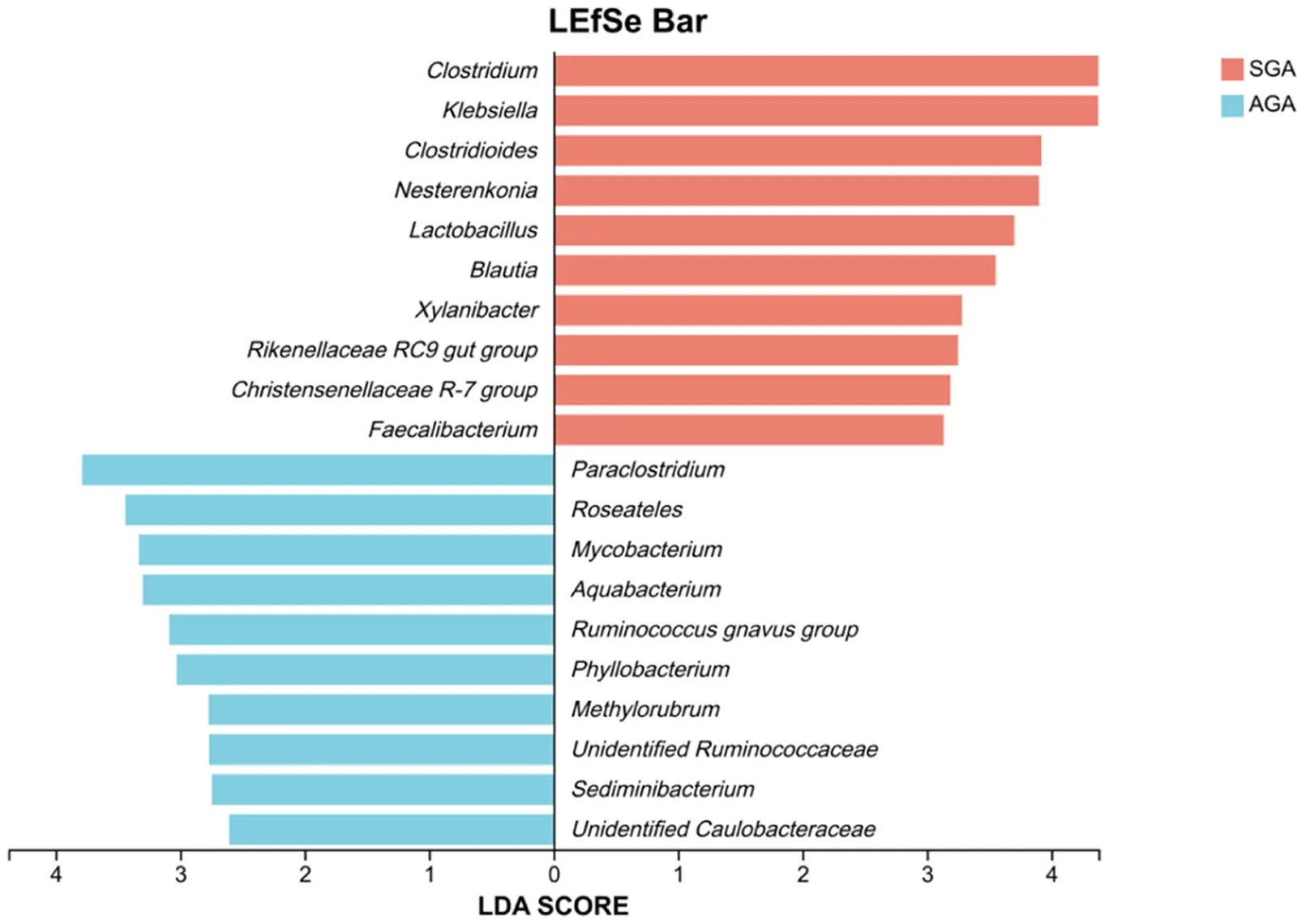
Differentially abundant genera between SGA and AGA groups identified by LEfSe analysis. LDA scores indicate the effect size of each differentially abundant taxon.

### 3.5 Correlation analysis of differential microbiota, metabolites, and clinical growth indicators between the two groups

#### 3.5.1 Correlation between differential metabolites and clinical growth indicators

By integrating partial Spearman correlation analysis (adjusting for gestational age, sex, and maternal delivery mode) with intergroup differential data, a total of 19 metabolites were identified that were significantly correlated with cord blood IGF-1, birth weight, or birth length (|r| > 0.3, *P* < 0.05). Among them, 16 metabolites were significantly downregulated in the SGA group (*P* < 0.05), primarily consisting of glycerophospholipids [e.g., Lpe(16:1), Lpe(22:5), Pc(18:2/0:0)], which are involved in cell membrane remodeling and energy metabolism. The bilirubin derivatives (4E,15Z)-Bilirubin and Lumirubin, along with the environmental pollutant Chlorobenzene, showed negative correlations with growth parameters and were significantly elevated in the SGA group. This suggests that their intrauterine accumulation may directly inhibit fetal growth or reflect immature bile metabolic function (Table 2 and Figure 7).

**Table 2:** Correlation between cord blood metabolites and clinical indicators.

Metabolite name	*r*-value	Associated clinical indicator	Change in SGA group	KEGG pathway
Lpe(18:1)	0.418	Weight	Down	Glycerophospholipid metabolism (map00564)
1-Nonadecanoyl-glycero-3-phosphoethanolamine	0.373	Weight	Down	Glycerophospholipid metabolism (map00564)
Pe(o-14:1/4:0)	0.369	Weight	Down	Glycerophospholipid metabolism (map00564)
Lpe(20:3)	0.357	Weight	Down	Glycerophospholipid metabolism (map00564)
Pc(o-20:4)	0.353	IGF-1	Down	Glycerophospholipid metabolism (map00564)
Lpe(22:5)	0.349	Weight	Down	Glycerophospholipid metabolism (map00564)
Pc(20:4/0:0)	0.346	IGF-1	Down	Glycerophospholipid metabolism (map00564)
Stearoyl lysophosphatidylethanolamine	0.341	IGF-1	Down	Glycerophospholipid metabolism (map00564)
Pc(20:3/0:0)	0.33	IGF-1	Down	Glycerophospholipid metabolism (map00564)
Lpc(20:4)	0.324	IGF-1	Down	Glycerophospholipid metabolism (map00564)
1-Heneicosanoyl-glycero-3-phosphoserine	0.32	IGF-1	Down	Glycerophospholipid metabolism (map00564)
Lpe(16:1)	0.319	Weight	Down	Glycerophospholipid metabolism (map00564)
Pc(22:6/0:0)	0.308	IGF-1	Down	Glycerophospholipid metabolism (map00564)
Pc(18:2/0:0)	0.306	IGF-1	Down	Glycerophospholipid metabolism (map00564)
Saxitoxin	0.305	Weight	Down	Neuroactive ligand-receptor interaction (map04080)
Pe(o-16:2/4:0)	0.301	IGF-1	Down	Glycerophospholipid metabolism (map00564)
Lumirubin	−0.316	IGF-1	Up	Bile secretion (map04976)
(4E,15Z)-Bilirubin	−0.331	Weight	Up	Bile secretion (map04976)
Chlorobenzene	−0.356	Weight	Up	ABC transporters (map02010)

Data are presented as partial Spearman correlation coefficients (r) adjusted for gestational age, sex, and maternal delivery mode. Positive r values indicate a positive correlation between metabolite levels and the respective clinical indicator, while negative r values indicate a negative correlation. “Up” or “Down” in the “Change in SGA Group” column refers to the relative abundance of the metabolite in the SGA group compared to the AGA group. All correlations met the threshold for FDR-adjusted *P* < 0.05. Rows are arranged by descending absolute correlation coefficient (|r|).

**Figure 7:**
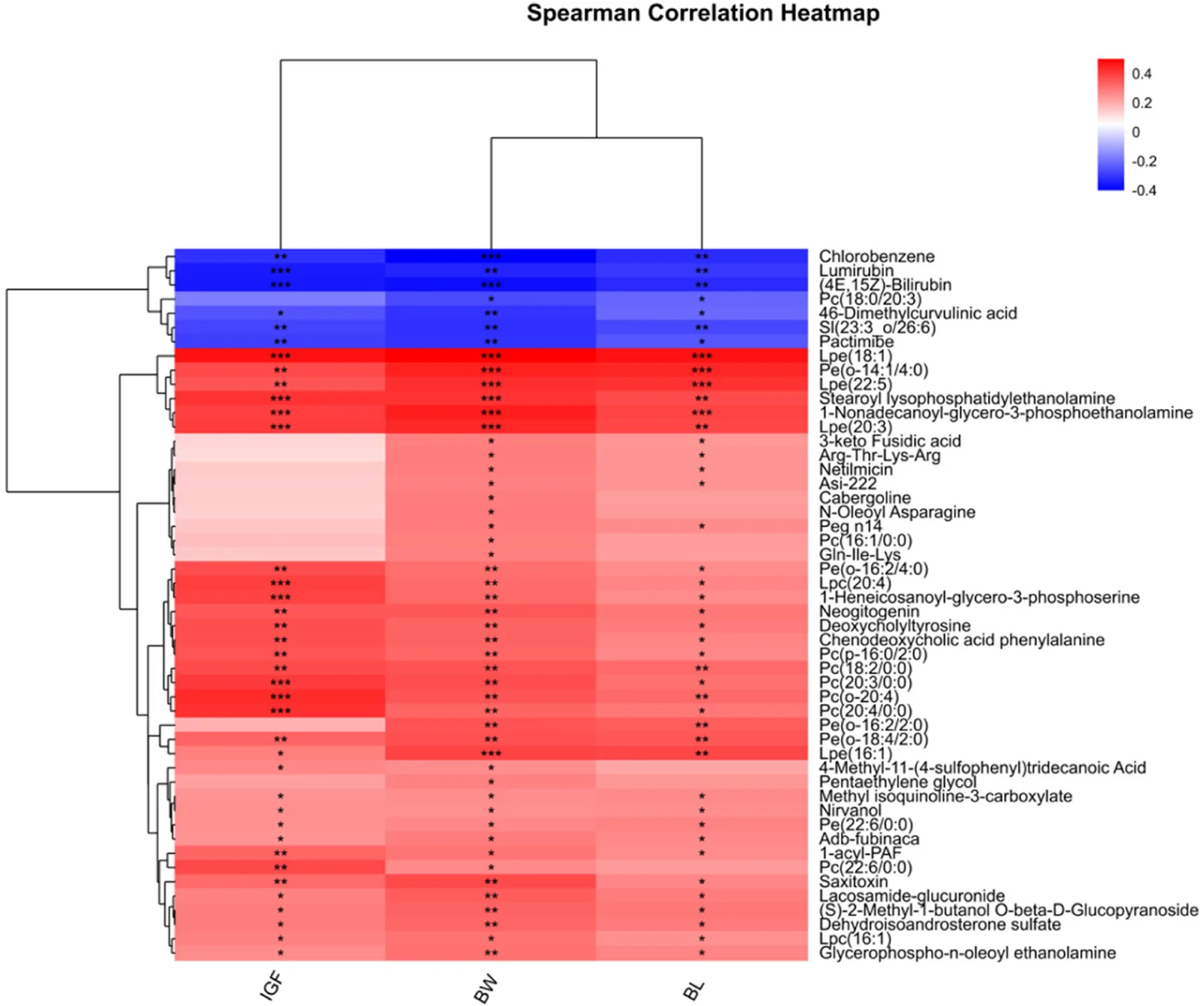
Correlation heatmap of cord blood metabolites and clinical features. The color bar on the right side of the heatmap represents the correlation coefficient (Corr. coef.), where red indicates positive correlation and blue indicates negative correlation. Asterisks in the cells denote the statistical significance level (*P* < 0.01, *P* < 0.001). BW, birth weight; BL, birth length.

#### 3.5.2 Correlation between differential microbiota and clinical growth indicators

Based on the integrated analysis of LEfSe intergroup differential analysis and partial Spearman correlation adjusting for gestational age, sex, and maternal delivery mode, a total of 12 genera were identified that exhibited a moderate correlation with at least one clinical growth indicator (birth weight or birth length) (|r| > 0.3, *P* < 0.05). Among them, *Hungatella* (*r* = −0.384, *P* < 0.05), *Christensenellaceae* R-7 group (*r* = −0.367, *P* < 0.05), unidentified *Lachnospiraceae* (*r* = −0.358, *P* < 0.05), and *Clostridium* (*r* = −0.356, *P* < 0.05) showed the most significant negative correlations with birth weight. *Clostridium* (*r* = −0.339, *P* < 0.05) and *Hungatella* (*r* = −0.312, *P* < 0.05) also exhibited significant negative correlations with birth length (Table 3). Cord blood IGF-1 levels showed a negative association trend with the relative abundances of the aforementioned 11 genera (r range: −0.046 to −0.205), with *Romboutsia* (*r* = −0.224) and *Faecalibacterium* (*r* = −0.240) demonstrating the most pronounced negative trends in the SGA group. Correspondingly, in the AGA group, genera positively correlated with growth indicators tended to show a positive association trend with IGF-1 levels. Although the absolute values of the correlation coefficients between these genera and IGF-1 were relatively low, the direction-specificity of the correlations between groups was notable (Figure 8).

**Table 3:** Correlation between gut microbiota and clinical indicators.

Genus name	r with IGF-1	r with birth weight	r with birth length	Group
*Phyllobacterium*	0.217	0.303	0.243	AGA
*Unidentified Oscillospiraceae clade*	−0.046	−0.300	−0.217	SGA
*Unidentified RF39 clade*	−0.076	−0.303	−0.194	SGA
*Blautia*	−0.204	−0.315	−0.277	SGA
*Unidentified Lachnospiraceae clade*	−0.105	−0.316	−0.271	SGA
*Agathobacter*	−0.143	−0.323	−0.260	SGA
*Alistipes*	−0.089	−0.339	−0.285	SGA
*Lachnospiraceae NK4A136 group*	−0.112	−0.351	−0.259	SGA
*Clostridium*	−0.205	−0.356	−0.339	SGA
*Unidentified Lachnospiraceae*	−0.148	−0.358	−0.247	SGA
*Christensenellaceae R-7 group*	−0.115	−0.367	−0.283	SGA
*Hungatella*	−0.110	−0.384	−0.312	SGA

Data are presented as partial Spearman correlation coefficients (r) adjusted for gestational age, sex, and maternal delivery mode. Rows are arranged by descending absolute correlation coefficient with birth weight. All correlations with birth weight and length met the threshold for moderate correlation (|r| > 0.3, FDR-adjusted *P* < 0.05). Negative values indicate an inverse association between microbial abundance and the respective clinical parameter.

**Figure 8:**
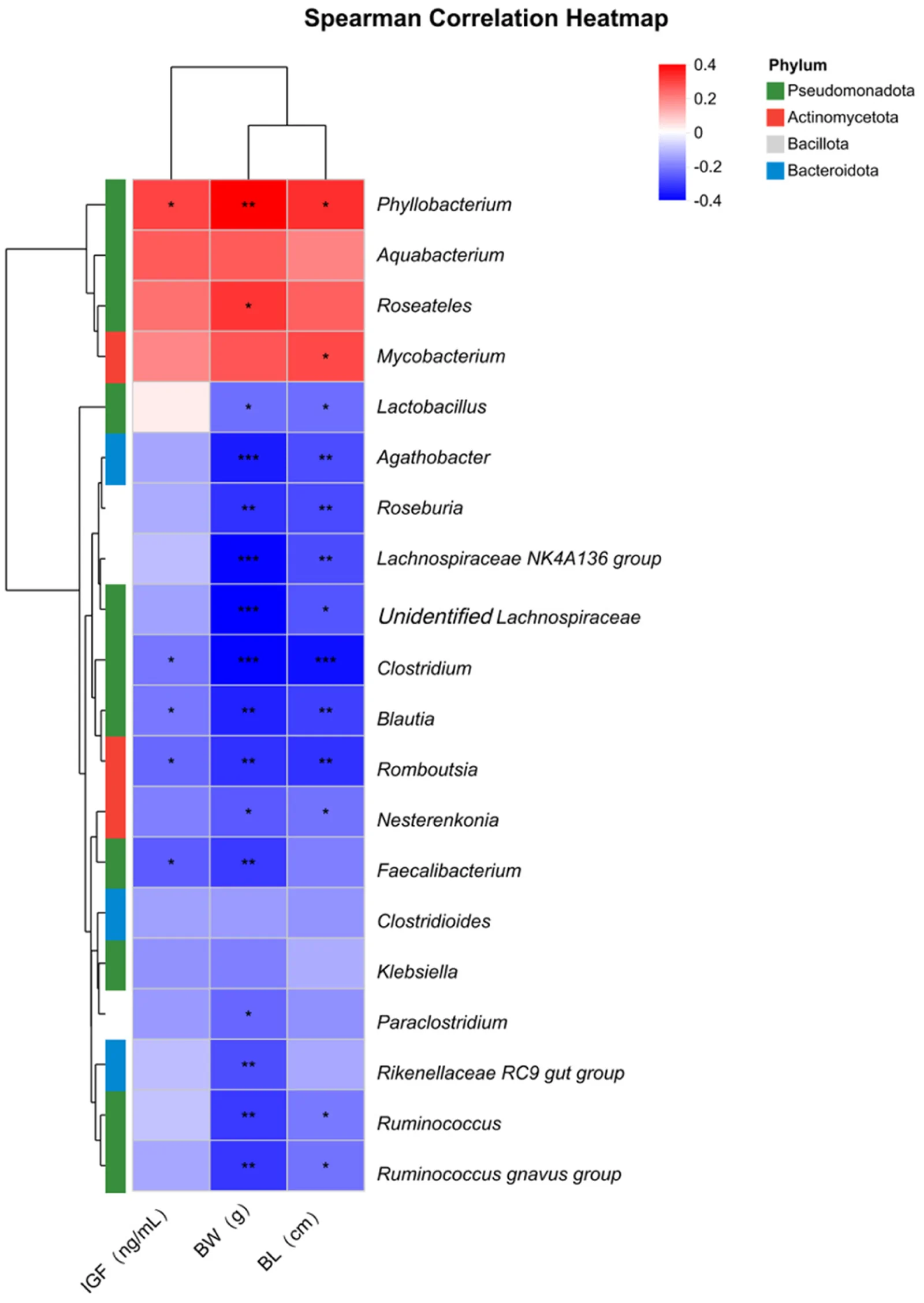
Heatmap of correlations between differential genera and clinical characteristics. The color scale on the right indicates the correlation coefficient, with red representing positive correlation and blue representing negative correlation. The colored blocks to the right of the bacterial taxa denote the corresponding phylum classification. Asterisks indicate statistical significance in the correlation test (*P* < 0.05). BW, birth weight; BL, birth length.

#### 3.5.3 Correlation between differential metabolites and gut microbiota

Based on Spearman correlation analysis, a total of 23 significant associations between cord blood metabolites and meconium microbiota were identified (|r| > 0.3, *P* < 0.05), as detailed in Table 4 and Figure 9. After excluding potential interference from background bacteria or environmental sources, this study found that the environmental pollutant chlorobenzene exhibited the clearest association pattern with gut microbiota, which was highly consistent with the clinical growth phenotypes. Correlation results showed that chlorobenzene was significantly positively correlated with *Clostridium*, *Alistipes, Lachnospiraceae* NK4A136 group, and *Christensenellaceae* R-7 group, suggesting that the intrauterine accumulation of chlorobenzene in the SGA group may exacerbate the disturbance of the early colonizing microbiota structure.

**Table 4:** Correlation between cord blood metabolites and gut microbiota.

Metabolite name	Genus name	*r*	*P*-value	FDR-adjusted P
Chlorobenzene	*Alistipes*	0.371	2.12 × 10⁻⁶	<0.001
Chlorobenzene	*Clostridium*	0.359	2.71 × 10⁻⁶	<0.001
Pc(p-16:0/2:0)	*Acidibacter*	0.352	5.17 × 10⁻⁶	<0.001
Chlorobenzene	*UCG-005*	0.336	2.83 × 10⁻⁵	<0.001
Chlorobenzene	*Lachnospiraceae NK4A136 group*	0.336	1.50 × 10⁻⁵	<0.001
Chlorobenzene	*Unidentified Ruminococcaceae*	0.329	3.94 × 10⁻⁵	<0.001
Chlorobenzene	*Unidentified Lachnospiraceae clade*	0.327	4.24 × 10⁻⁵	<0.001
(S)-2-Methyl-1-butanol O-beta-D-Glucopyranoside	*Methylorubrum*	0.321	3.41 × 10⁻⁵	<0.001
Lpc(16:1)	*Acidibacter*	0.32	3.46 × 10⁻⁵	<0.001
Glycerophospho-n-oleoyl ethanolamine	*Acidibacter*	0.318	3.94 × 10⁻⁵	<0.001
Chlorobenzene	*Unidentified Lachnospiraceae*	0.317	2.58 × 10⁻⁶	<0.001
Chlorobenzene	*Unidentified Clostridia UCG-014*	0.309	9.20 × 10⁻⁵	<0.001
Chlorobenzene	*Christensenellaceae R-7 group*	0.309	9.46 × 10⁻⁵	<0.001
Chlorobenzene	*Unidentified Prevotellaceae*	0.303	1.59 × 10⁻⁴	<0.001
Chlorobenzene	*Methylorubrum*	−0.306	7.63 × 10⁻⁵	<0.001
Saxitoxin	*NK4A214 group*	−0.307	1.32 × 10⁻⁴	<0.001
Arg-Thr-Lys-Arg	*UCG-005*	−0.307	1.32 × 10⁻⁴	<0.001
Saxitoxin	*UCG-005*	−0.312	1.00 × 10⁻⁴	<0.001
Chlorobenzene	*Mycobacterium*	−0.317	5.68 × 10⁻⁵	<0.001
Chlorobenzene	*Dechloromonas*	−0.345	1.10 × 10⁻⁵	<0.001
Chlorobenzene	*Phyllobacterium*	−0.353	4.23 × 10⁻⁶	<0.001
Chlorobenzene	*Sediminibacterium*	−0.354	5.67 × 10⁻⁶	<0.001
Chlorobenzene	*Acidibacter*	−0.368	1.94 × 10⁻⁶	<0.001

Correlations were analyzed by partial Spearman's rank test adjusting for gestational age, and maternal delivery mode, with FDR correction. Rows are arranged by descending absolute correlation coefficient (|r|). Positive r values indicate concomitant increase between metabolite levels and bacterial relative abundance; negative r values indicate an inverse relationship.

**Figure 9:**
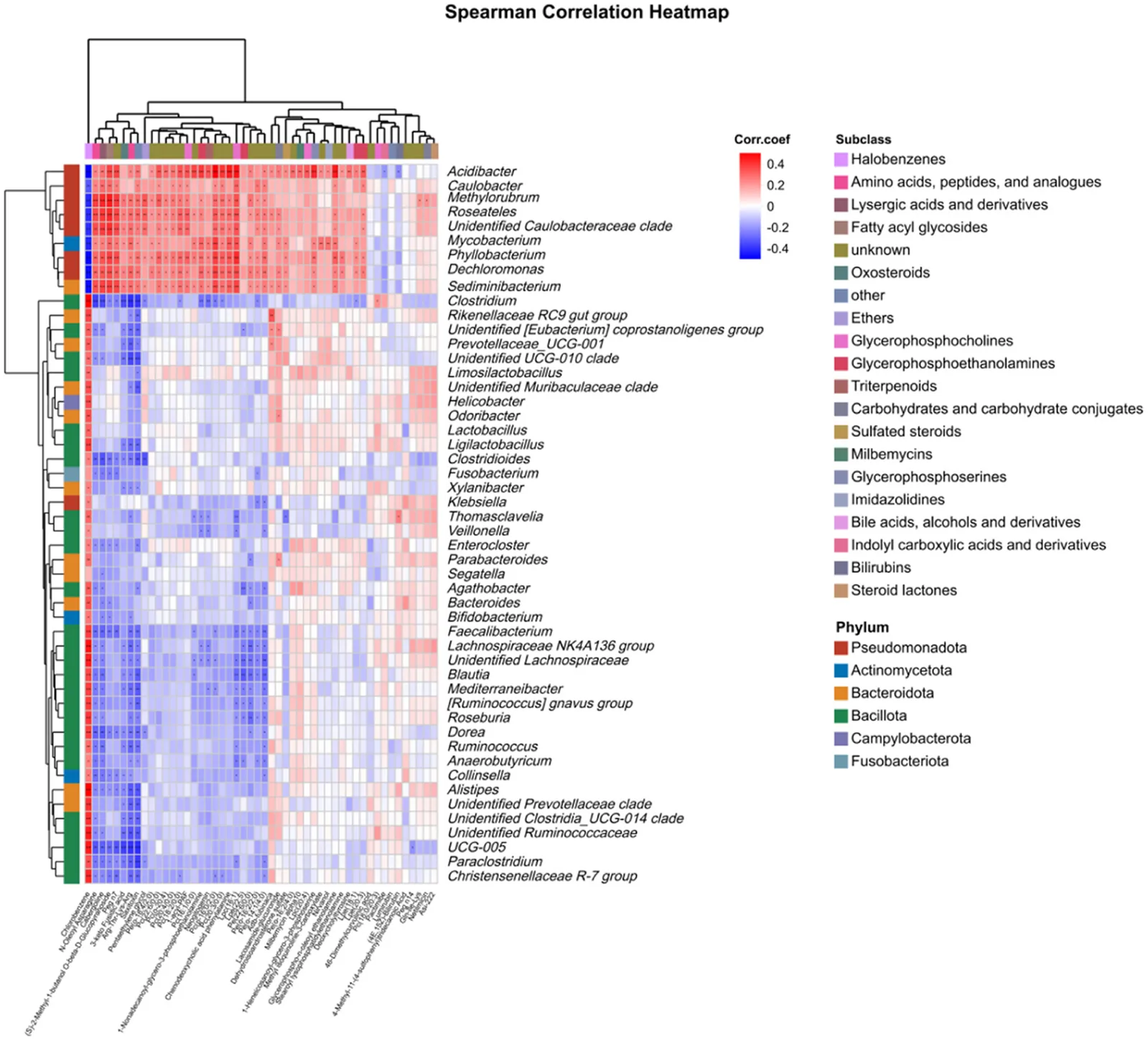
Heatmap of correlations between cord blood metabolites and gut microbiota. Metabolites are color-coded by chemical subclass. The color bar on the right indicates the correlation coefficient. The color bar on the left denotes bacterial phyla.

## 4 Discussion

This study systematically compared the differences in endocrine status, intrauterine metabolic profile characteristics, and early postnatal gut-colonizing microbiota between SGA and AGA newborns, and revealed that against the backdrop of intrauterine growth restriction, SGA newborns not only exhibited downregulated cord blood IGF-1 levels and systemic metabolic profile disturbances, but also displayed characteristic alterations in the structure and composition of early meconium microbiota. Further correlation analysis identified significant associations between differential metabolites, characteristic microbiota, birth anthropometric parameters, and IGF-1 levels, suggesting that alterations in the intrauterine metabolic environment are associated with abnormal colonization patterns of early gut microbiota in SGA newborns.

IGF-1 is a core endocrine factor regulating fetal intrauterine growth and development, participating in multiple physiological processes including cell proliferation and differentiation, tissue and organ development, and maintenance of energy metabolic homeostasis. Cord blood IGF-1 levels can objectively reflect fetal intrauterine nutritional reserves and growth potential (17, 18). The results of this study once again verified the significantly reduced cord blood IGF-1 levels in SGA newborns and their positive correlation with birth weight and length (19, 20), confirming that the study population exhibited a typical endocrine phenotype of intrauterine growth restriction. However, the moderate partial correlation coefficients indicate that IGF-1 explains only a modest proportion of the variance in fetal growth parameters. The majority of variance is attributable to other genetic, nutritional, and environmental factors, underscoring the multifactorial nature of intrauterine growth regulation. This provides a phenotypic basis for further dissecting the intrinsic association between metabolic reprogramming and gut microbiota dysbiosis.

Glycerophospholipids are core structural components of the phospholipid bilayer in eukaryotic cell membranes and act as second messenger precursors to participate in key physiological processes such as cell cycle regulation, inflammatory response, and membrane transport. Metabolic dysregulation of glycerophospholipids can lead to abnormal fetal intrauterine development (21, 22). This study found that lipid and lipid-like molecules in cord blood of SGA newborns, particularly glycerophospholipid compounds including phosphatidylcholine (PC), lysophosphatidylcholine (LPC), and lysophosphatidylethanolamine (LPE), were significantly downregulated in SGA cord blood, which is consistent with the results of a previous systematic review. That review reported that LysoPC (16:1) levels were uniformly downregulated across six included studies on cord blood of fetal growth restriction (FGR)/SGA newborns, revealing that phospholipid metabolic dysregulation is a key pathological link in fetal growth restriction (23). Further correlation analysis showed that the levels of these glycerophospholipid metabolites were significantly positively correlated with birth weight, birth length, and cord blood IGF-1 concentrations. Meanwhile, KEGG enrichment analysis revealed a significant difference in the choline metabolism pathway. As choline serves as a key rate-limiting precursor in the *de novo* synthesis pathway of glycerophospholipids, these findings suggest that SGA fetuses are associated with insufficient substrate supply for phospholipid synthesis and downregulated activity of the synthesis pathway, thereby restricting the rate of cell membrane biosynthesis and impairing fetal intrauterine growth. Previous studies have shown that IGF-1 is involved in phospholipid synthesis via the PI3K/Akt signaling pathway, although the precise mechanism in fetal growth restriction remains to be fully elucidated (24). Observational data from this study suggest that reduced IGF-1 levels in SGA are associated with insufficient anabolic signals mediated by IGF-1, further suppressing phospholipid biosynthesis. Conversely, phospholipid deficiency weakens cellular responsiveness to IGF-1, forming a negative regulatory loop at the metabolic and endocrine levels (24, 25).

The association between intrauterine exposure to environmental pollutants and fetal growth restriction has garnered increasing attention. Existing studies have confirmed that maternal exposure during pregnancy to various environmental chemicals—such as per- and polyfluoroalkyl substances (PFAS), phenols, and polycyclic aromatic hydrocarbons (PAHs)—is associated with an increased risk of delivering small-for-gestational-age (SGA) infants (26, 27). Consistent with these findings, the present study also identified significantly elevated levels of the environmental pollutant chlorobenzene in the cord blood of SGA newborns, which showed a significant negative correlation with birth weight, birth length, and IGF-1 levels. This observational pattern suggests an association between elevated chlorobenzene signal and SGA status. However, because chlorobenzene is a common solvent and chromatographic contaminant, this finding requires rigorous validation against extraction blanks and quality control samples to exclude analytical artifacts before concluding that it represents genuine in utero exposure. If confirmed as a genuine exposure, its association with endogenous metabolic dysregulation might contribute collectively to understanding fetal growth restriction.

Chlorobenzene belongs to the class of halogenated aromatic hydrocarbon persistent organic pollutants. As a lipophilic organic pollutant, it exhibits high placental permeability, allowing it to freely traverse the placental barrier and accumulate continuously in the fetal circulation (28). Current toxicological research indicates that chlorobenzene can hinder embryonic cell proliferation and organ differentiation through multiple mechanisms, including inducing oxidative stress damage and obstructing osteoblast differentiation (29, 30). Direct evidence for chlorobenzene-specific transplacental transfer in humans remains limited, and the cited toxicological studies employed higher-dose animal models; thus, extrapolation to human intrauterine exposure requires caution. The findings of this study imply that intrauterine exposure to exogenous environmental pollutants may represent a non-nutritive risk factor for the occurrence of SGA. If validated, its synergistic interaction with endogenous metabolic dysregulation likely contributes collectively to the initiation and progression of fetal growth restriction.

Early-life gut microbiota colonization is a critical component of perinatal developmental programming. To characterize the early microecological features of SGA newborns, this study performed 16S rRNA sequencing analysis on meconium samples collected within 48 h after birth. The results showed no significant differences in alpha diversity between the SGA and AGA groups; however, beta diversity analysis revealed a significant separation in the overall microbial community structure between the two groups. This finding is consistent with the results reported by Chen et al. in their study on term SGA infants (15). Furthermore, Chang et al., in a study involving very-low-birth-weight preterm infants, observed that although no significant differences were found in alpha or beta diversity at birth between SGA and AGA groups, a significant separation in beta diversity emerged by day 30 of life (31). This indicates that alterations in the gut microbiota of SGA infants may not involve changes in the quantity of early colonizers, but rather specific modifications in the structural composition of the microbial community.

At the compositional level, the initial gut microbiota of SGA newborns exhibited characteristic alterations at the genus level. The abundance of facultative anaerobes representing early colonizers (such as *Escherichia-Shigella* and *Enterococcus*) showed a decreasing trend in the SGA group, whereas strict anaerobes and conditional pathogens typically appearing during later maturation (such as *Klebsiella* and *Clostridium*) were significantly increased. This colonization pattern, which resembles the dysbiotic microbiota profile observed in preterm infants, likely reflects insufficient intestinal motility or immature mucosal immune development during the fetal period in SGA infants (32, 33). LEfSe analysis further confirmed that genera such as *Clostridium, Clostridioides, Klebsiella, Nesterenkonia, Blautia*, and *Faecalibacterium* were significantly enriched in the SGA group. Although these taxa can appear as contaminants in low-biomass samples, their enrichment pattern was consistent with the SGA phenotype and distinct from environmental controls, supporting their biological origin in meconium. Escherichia coli (within *Escherichia-Shigella*) and Enterococcus are core early-colonizing facultative anaerobes in the neonatal gut. They consume residual oxygen within the intestine, thereby lowering the redox potential and laying the foundation for the subsequent colonization of obligate anaerobes and the establishment of gut homeostasis (34). In this study, the decline in early colonizers like *Escherichia* and *Enterococcus* in the SGA group, coupled with the premature colonization of late-maturing bacteria from the phylum Bacillota—such as *Clostridium, Blautia*, and *Faecalibacterium*—indicates a potential imbalance in the temporal sequence of early microbiota colonization and structural dysbiosis in SGA newborns.

Furthermore, this study found a significant enrichment of core short-chain fatty acid (SCFA)-producing bacteria, including *Clostridium, Blautia*, and *Faecalibacterium*, in the SGA group. This phenomenon can be attributed not only to the disturbance in early gut microbiota colonization structure but may also be closely related to postnatal catch-up growth in SGA infants. Durda-Masny et al. (35) demonstrated in a prospective study that SGA children undergoing catch-up growth exhibited accelerated gut microbiota succession and increased diversity. Their study also found elevated abundances of SCFA-producing and obesity-promoting bacteria during catch-up growth. The premature enrichment of SCFA-producing bacteria in the gut microbiota of SGA infants may represent an adaptive response by the body to compensate for insufficient growth by increasing energy extraction from the diet. However, this phenomenon might also contribute to an increased risk of future obesity.

Correlation analysis between intergroup differential genera and clinical characteristics revealed that genera enriched in the SGA group—such as *Clostridium, Hungatella*, and *Christensenellaceae*_R-7_group—exhibited a moderate negative correlation with birth weight or length. After adjustment for delivery mode, sex and gestational age using partial Spearman correlation, these negative associations remained significant (*p* < 0.05). Overall, these genera showed a negative association trend with cord blood IGF-1 levels, whereas the majority of gut microbiota in the AGA group displayed positive correlations with birth length, weight, and IGF-1, demonstrating clear group specificity. Recent studies have suggested that the host's endocrine status can also modulate intestinal mucosal immunity and microbiota colonization patterns (36). Low IGF-1 levels have been hypothesized to increase the risk of pathogen colonization by compromising the intestinal immune barrier function, thereby altering microbiota composition (37). IGF-1 is a key factor in maintaining the proliferation, differentiation, and repair of intestinal epithelial cells. Observational and experimental data suggest that its deficiency is associated with impaired integrity of the intestinal mucosal barrier and increased permeability. This may render the intestine more susceptible to invasion by pathogens and their toxins (e.g., lipopolysaccharide), triggering local inflammatory responses. Consequently, this disrupts normal microbial niches, promotes the overgrowth of pathogenic bacteria, and inhibits the colonization of beneficial bacteria (38). Although the absolute values of the correlation coefficients between SGA-enriched genera and cord blood IGF-1 observed in this study were relatively low, this may suggest that the regulatory effect of IGF-1 on the microbiota is largely indirect. IGF-1 may select for colonizing bacteria by maintaining the integrity of the intestinal mucosal barrier, rather than directly exerting a linear regulatory effect on microbiota changes. Furthermore, the high functional redundancy of the gut microbiota may allow the microenvironmental alterations induced by low IGF-1 to be synergistically responded to by multiple genera, thereby diluting the signal from any single genus.

Beyond endocrine regulation, alterations in the intrauterine metabolic environment may also be associated with early postnatal microbiota colonization patterns (39, 40). Correlation analysis between cord blood metabolites and the gut microbiota revealed that cord blood chlorobenzene concentrations were significantly positively correlated with the abundances of genera enriched in the SGA group, including *Clostridium, Alistipes, Lachnospiraceae*_NK4A136_group, and *Christensenellaceae*_R-7_group. This association suggests that, if validated as a genuine exposure, intrauterine accumulation of chlorobenzene might be associated with the structural shift in the gut microbiota of SGA newborns.

As a hypothesis requiring experimental validation, chlorobenzene might cross the placental barrier into fetal circulation and be excreted into the intestine via bile or enterohepatic circulation. Through differential cytotoxicity and metabolic stress, it might exert a selective pressure on the colonizing microbiota, favoring anaerobic conditional pathogens with greater environmental tolerance (9, 37). If so, these organisms could be better adapted to the microenvironment under pollutant exposure, thereby gaining a colonization advantage, which could lead to disturbances in the temporal sequence and structural shifts in the microbiota. Concurrently, as we previously observed, the directional association of cord blood chlorobenzene levels with clinical growth indicators and IGF-1 was consistent (both negative). This observational pattern suggests that, if chlorobenzene is confirmed as a genuine intrauterine exposure, chlorobenzene-mediated dysbiosis might serve as a crucial link connecting intrauterine environmental exposure to difficulties in postnatal catch-up growth. This finding underscores the importance of considering the impact of environmental chemical exposures on early-life microecological programming in the etiological exploration of SGA.

Subsequently, by integrating cord blood IGF-1 levels, untargeted metabolomics, and meconium gut microbiota sequencing data, this study systematically revealed multilayered associations in SGA newborns: downregulated cord blood IGF-1 levels, metabolomic patterns centered on glycerophospholipid depletion, elevated signal of the environmental pollutant chlorobenzene, and imbalances in the temporal sequence and structural disturbances of gut microbiota colonization. Cross-sectional correlation analysis suggested that the low IGF-1 endocrine environment and intrauterine metabolic abnormalities are associated with abnormal colonization patterns of early gut microbiota in SGA infants.

However, this study has certain limitations. It adopted a cross-sectional design, which could only observe associations between variables but failed to establish causal temporality. The consecutive enrollment from a single center during a defined timeframe may limit generalizability to other geographic regions or ethnic populations. Additionally, We did not conduct functional experiments to verify the specific molecular mechanisms underlying the interaction between metabolites and the gut microbiota. Future studies should conduct large-sample prospective cohort studies to dynamically track intrauterine metabolic exposures, gut microbiota succession, and child growth and development trajectories.

## 5 Conclusions

SGA newborns exhibit downregulated IGF-1 endocrine levels, disturbances in the intrauterine metabolic profile, and abnormalities in gut microbiota structure and colonization timing at birth. Observational data from this cross-sectional study suggest that alterations in the intrauterine metabolic environment are associated with abnormal colonization patterns of the early gut microbiota in SGA infants. However, causal relationships cannot be established, and the chlorobenzene finding requires independent validation.

## Data Availability

The raw data supporting the conclusions of this article will be made available by the authors, without undue reservation.
